# Roles of children and their parents in the reduction of radiation risk after the 2011 Fukushima Daiichi Nuclear Power Plant accident

**DOI:** 10.1371/journal.pone.0188906

**Published:** 2017-12-13

**Authors:** Maya Sophia Fujimura, Yukako Komasa, Shinzo Kimura, Akira Shibanuma, Akiko Kitamura, Masamine Jimba

**Affiliations:** 1 Department of Community and Global Health, Graduate School of Medicine, The University of Tokyo, Tokyo, Japan; 2 Laboratory of International Epidemiology, Center for International Cooperation, Dokkyo Medical University, Tochigi, Japan; Northwestern University Feinberg School of Medicine, UNITED STATES

## Abstract

**Introduction:**

On March 11, 2011, Japan experienced its largest recorded earthquake with a magnitude of 9.0. The resulting tsunami caused massive damage to the Fukushima Daiichi Nuclear Power Plant reactors, and the surrounding environment was contaminated with radioactive materials. During this period, some residents were exposed to high levels of radiation (up to 5 millisieverts [mSv]), but since then, many residents have been exposed to low levels of radiation (<1 mSv). This study was conducted to assess the effects of lifestyle and attitude factors on external radiation exposure among Fukushima residents.

**Methods:**

This community-based, cross-sectional study was conducted in Nihonmatsu City of the Fukushima Prefecture from May to July 2014. The population survey targeted 6,884 children between the ages of 0–15 years, and a personal radiation badge and questionnaire were administered to each of the residences. Multiple linear regression analysis was used to assess the impact of lifestyle and attitude factors on external radiation dose.

**Results:**

The study participants (population size [n] = 4,571) had an additional mean radiation dose of 0.65 mSv/year, which is small as compared to the mean radiation dose 6 months after the disaster (1.5 mSv/year), in 2012 (1.5 mSv/year), and in 2013 (1.0 mSv/year). External radiation doses statistically varied by socio-demographic and lifestyle factors. Participants living in wooden residences (*p*-value<0.001) and within 100 meters of a forest (*p* = 0.001) had higher radiation exposure. Conversely, participants with a cautious attitude towards radiation had lower radiation exposure (beta [b] = -0.124, *p* = 0.003).

**Conclusion:**

Having a cautious attitude towards radiation and being aware of exposure risks proved to be significant in the reduction of external radiation dose. Therefore, in the event of future radiation disasters, attitudes towards and awareness of radiation should be considered in the reduction of exposure risk and implementation of radiation protection.

## Introduction

### Nuclear power plant accident in Fukushima, Japan

On March 11, 2011, Japan experienced its largest recorded earthquake with a magnitude of 9.0 [1]. The earthquake and resulting tsunami caused massive damage to the Fukushima Daiichi Nuclear Power Plant (FDNPP) reactors, which released radioactive material, such as Cesium-134 and Cesium-137, into the surrounding environment. The incident caused a series of nuclear accidents lasting between March 12, 2011 and March 15, 2011 [2, 3]. In addition, on March 25, residents within 20 to 30 kilometers of the radioactive- site were urged to evacuate [4]. The FDNPP incident was the largest nuclear accident to occur since the Chernobyl disaster of 1986 [5]. During this period, some residents in the evacuation zone were exposed to high levels of radiation (up to 5 millisieverts [mSv]), but since then, many residents living in the surrounding areas, such as Fukushima City, have been exposed to low levels of radiation (<1 mSv) [6, 7]. While much of the radiation has been removed from the physical environment, residents living within the Fukushima Prefecture are still plagued with mental, social, and environmental consequences, such as relocating out of the region or moving into temporary housing [8, 9].

### Health effects of radiation

Exposure to radiation increases the risk of many long-term health effects, such as thyroid cancer, leukemia, and cardiovascular disease [10–12]. Even low dose radiation exposure, which is currently present in the Fukushima Prefecture, has been shown to increase the risk of serious disease. While cancer has been reported to be one of the main risks of radiation exposure, a definitive causal relationship has not been established as a result of confounding variables, such as subject age, sex, and lifestyle factors, including tobacco use [13].

In addition, as low dose radiation spreads through a contaminated environment, the average radiation doses from natural resources within the environment also fluctuate. During the 2011 FDNPP accident, the average radiation dose from natural resources was 0.09 mSv/h (average of 15 control badges). By 2014, the estimated natural resource radiation dose was 0.06 mSv/h (average of 20 control badges). The Radiation Medical Science Center of Fukushima Medical University recommended conducting further studies to investigate the relationship between lifestyle and behavioral factors and radiation exposure levels to better understand public health risks and concerns [14]. These health risks are not limited to only physical concerns, but include mental and social issues as well. For example, in the 1986 Chernobyl nuclear disaster and the 1979 Three Mile Island nuclear disaster, the most commonly cited issue was long-term psychological effects. Further, the Fukushima Mental Health Survey found that Fukushima evacuees experienced adverse mental health effects, including post-traumatic stress disorder (PTSD) and anxiety [15]. Thus, the current mental health of children living within this region should be further examined to prevent future adversities, such as bullying, discrimination, and stigmatization [16, 17].

### Reducing radiation risk

Detailed and continuous education on the risks of radiation must be provided to susceptible populations to increase radiation awareness and reduce exposure risk [18]. A large fraction of the Japanese general population, including students, has not been properly educated on the mechanisms and risks of radiation, with most individuals gathering information from biased news outlets [19]. Therefore, schools play an important role in the spreading of unbiased radiation information and accurate risk awareness [20, 21].

In addition, an individual’s understanding of radiation has been shown to be related to personal perceptions and environmental factors. While healthcare providers often have basic knowledge of radiation, teachers tend to not educate students about radiation because the topic is deemed unimportant or inapplicable [22, 23]. In the Fukushima disaster, the lack of radiation knowledge and awareness led to a lack of fear for entering potentially harmful areas. Yet, proper radiation knowledge and awareness has also contributed to higher levels of anxiety among individuals living within a post-disaster setting [24]. If schools provide a proper education on radiation risks, students will become more knowledgeable about protection methods and potential long-term health effects.

### Objectives of the study

This study was conducted to assess the effects of lifestyle and attitude factors on external radiation exposure among Fukushima residents.

## Materials and methods

### Study population

**Fig 1:**
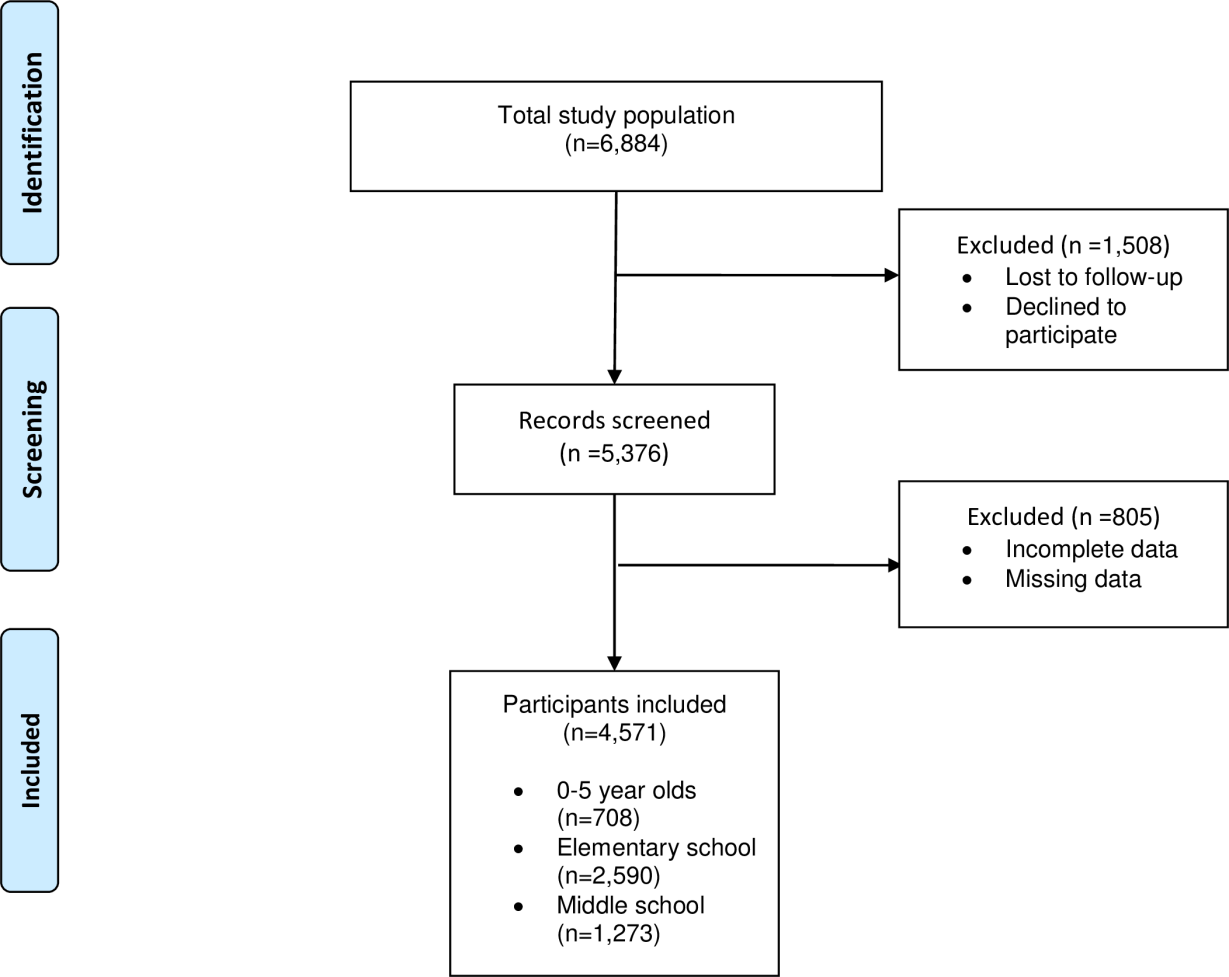
Flow chart of the study participants.

Nihonmatsu City is located within the Fukushima Prefecture and is 37–60 kilometers from the FDNPP. This study area is unique because it has retained the highest radiation dosage of the all the surrounding areas that were not instructed to evacuate [25]. Given the emergency disaster situation, a population survey was conducted among the residents of Nihonmatsu City. The population survey targeted 6,884 children and students between the ages of 0 and 15 years who lived in the study area. A total of 5,376 children participated in the study after informed consent was obtained from their guardian. After incomplete or missing data entries were removed, the remaining study population consisted of 4,571 participants, with 708 participants between the ages of 0 and 5 years, 2,590 participants of elementary school age, and 1,273 participants of middle school age (Fig 1).

### Study design and procedure

This community-based, cross-sectional study was conducted in Nihonmatsu City of the Fukushima Prefecture from May to July 2014. A personal radiation badge (Nagase-Landauer Ltd., Qulxel Badge) and questionnaire concerning lifestyle and attitude characteristics toward nuclear radiation were distributed to each residence with a child between the ages of 0 and 15 years. The research was conducted in collaboration with the city health promotion division, school education board, and regional universities, such as Dokkyo Medical University and The University of Tokyo.

The city district health promotion division distributed survey packages to each targeted residence through the school system. The survey packages contained a personal radiation badge, a pouch for the badge, a study information sheet, an informed consent form, a questionnaire, and a self-addressed return envelope. The personal radiation badges were used to monitor the radiation exposure of each individual. The badge could measure a total dose range of 0.01 mSv to 10 mSv. The background environmental dose was adjusted for the analysis of this study. For all study participants, the questionnaires were completed by their guardians, with the exception of the middle school students, who were instructed to complete the first half of the questionnaire on lifestyle factors by themselves. The questionnaires were returned using the self-addressed envelopes, and the personal radiation badges were collected at their respective schools at the end of the 2-month study period.

For the study follow-up, results were mailed back to the participating households, reporting the average external radiation dosage of each student from each school with possible reasons for variances provided. In addition, the results included information and advice from the city radiation specialist and associate professor of Dokkyo Medical University (SK) on food safety, air contamination, outdoor exposure, and radiation measurement and monitoring. The results also provided the phone number to the city health promotion division office for individuals who desired further internal and/or external radiation advice.

### Radiation exposure measurements

In the present study, the outcome variable was the radiation dose collected over the 2-month study period. The outcome was later calculated for the 1-year rate of radiation exposure in mSv. The external radiation dosage was measured for each child, and the guardians were responsible for completing the questionnaire.

The self-administered questionnaire consisted of three sections: socio-demographic and household characteristics, lifestyle, and attitude towards radiation. The socio-demographic and household characteristics measured the following factors: age, sex, and type of residence. For the type of residence, participants identified whether it was a house or apartment and whether it was constructed of wood, steel, or concrete [26]. Lifestyle factors were determined through the modifiable and unmodifiable factors of an individual’s life. The survey examined the daily activities and environmental and residential situation of each child. Factors included which floor of the house the child’s bedroom was located, if firewood was utilized in the home, what extracurricular activities the child participated in at school and the community, and if there was a forest within 100 meters of the child’s residence [27]. The attitude section consisted of eight questions regarding the guardian’s attitude towards radiation. Six yes/no questions measured the awareness and caution exercised by each guardian living in an area with low dose radiation, and two multiple choices questions measured the guardian’s level of cautiousness and concern towards radiation [28].

During the study period, the environmental air radiation dose rates varied among residence areas. The first floor of residences had an air radiation dose rate between 0.27–0.35 mSv/h when measured 1 m from the floor; however, when measured 0 cm from the floor, the air radiation dose rate was between 0.18–0.36 mSv/h. The second floor of residences had an air radiation dose rate between 0.31–0.34 mSv/h when measured 1 m from the floor; when measured 0 cm from the floor, the air radiation dose rate was between 0.25–0.28 mSv/h. In addition, the wall closest and the wall opposite to the decontaminated forest had an air radiation dose rate between 0.29 mSv/h and 0.23 mSv/h, respectively, when measured 1 m from the ground of the residence. Based on the monitoring survey, the forest air radiation dose rates were between 0.92–1.01 mSv/h, when measured 1 m from the ground, and between 1.03–1.06 mSv/h when measured 50 cm from the ground.

### Data collection and analysis

This study used external radiation measurements collected 3 years after the 2011 FDNPP accident. Data were collected from each participant’s personal radiation badge, which they wore for a 2-month period, from May 16, 2014, to July 15, 2014. The personal radiation badge measured the equivalent external exposure of radiation adsorbed by the individual. The personal radiation badges (Nagase-Landauer Ltd., Qulxel Badge) could measure a total dose range of 0.01 mSv to 10 mSv (precision and error estimate of ± 7% at 0.1 mSv and within ± 3% at 1 mSv or higher). Personal radiation badges were distributed to each household and worn by the children. Each badge contained an identification bar code that was later linked with the corresponding questionnaire.

All data were analyzed using Statistical Package for Social Science version 23 (SPSS 23). Data with missing or incomplete information were excluded from the analyses. Independent sample t-tests were used to analyze the relationship between the mean radiation dose and each independent variable: socio-demographic and household characteristics, lifestyle factors, and attitude factors. One-way analysis of variance (ANOVA) was used to assess the relationship between external radiation exposure and types of residence, bedroom floors, decontamination status, level of cautiousness and concern towards radiation. Multiple linear regression was conducted to examine the association between all variables of lifestyle and attitude factors and external radiation exposure. The statistical significance was set at 5%. Multicollinearity was assessed using variance inflation factor (VIF), and multicollinearity was considered when the VIF value exceeded 2.5. However, no multicollinearity was detected.

### Ethical considerations

This study was approved by the Research Ethics Committee of the Graduate School of Medicine at the University of Tokyo in Japan. Ethical approval was also obtained from the Dokkyo Medical University. Finally, the Nihonmatsu City school board and the city health promotion division reviewed and approved the study.

## Results

Data were analyzed by stratified age groups. The study participants (population size [n] = 4,571) had an additional mean radiation dose of 0.65 mSv/year, which is small as compared to the mean radiation dose 6 months after the disaster (1.5 mSv/year), in 2012 (1.5 mSv/year), and in 2013 (1.0 mSv/year). By stratified age group, participants between the ages of 0–5 years (n = 708), elementary school students (n = 2,590), and middle school students (n = 1,273) had mean radiation doses of 0.65 mSv/year (standard deviation [SD] 0.25), 0.65 mSv/year (SD 0.27), and 0.64 mSv/year (SD 0.27), respectively. Since 2011, the estimated background environmental radiation has ranged from 0.07–0.09 mSv/h. However, during the present study period, the average environmental background radiation was 0.06 mSv/h (average of 20 control badges).

Table 1 shows the socio-demographic characteristics and types of residences by age categories. Wooden houses were the most common residence types across all three age groups: 477 participants of the 0-5-year-old group (77.9%), 1,980 participants of the elementary school group (81.2%), and 1,002 participants of the middle school group (87.1%) lived in wooden residences. For all age groups, residence material was associated with external radiation dose. The mean external radiation doses differed within each age group by the type of residence material: the 0-5-year-old group (*p*<0.001), the elementary school group (*p*<0.001), and the middle school group (*p*<0.001). Wooden residences were found to have the highest mean radiation exposure rate, as compared to steel and concrete residences. However, the mean radiation doses across all three age groups remained insignificant (*p*>0.5).

**Table 1:** Comparison of mean radiation doses across socio-demographic and household factors (n = 4,571).

			n	%	Mean (mSv)	SD	*p*-value
**Age (n = 4,570)**		0-5-year-olds	708		0.65	0.25	
		Elementary school	2,590		0.66	0.27	
		Middle school	1,272		0.64	0.27	
**Sex (n = 4,449)**	0-5-year-olds	Male	344	49.2	0.66	0.26	0.330
		Female	355	50.8	0.64	0.24	
	Elementary school	Male	1,289	51.0	0.65	0.29	0.777
		Female	1,238	49.0	0.65	0.24	
	Middle school	Male	614	50.2	0.65	0.29	0.201
		Female	609	49.8	0.63	0.25	
**Type of residence (n = 4,199)**	0-5-year-olds	Wooden	519	84.8	0.68	0.26	<0.001
		Steel	46	7.5	0.54	0.18	
		Concrete	47	7.6	0.52	0.20	
	Elementary school	Wooden	2125	87.1	0.67	0.27	<0.001
		Steel	155	6.3	0.54	0.17	
		Concrete	157	6.4	0.52	0.24	
	Middle school	Wooden	1053	91.5	0.66	0.26	<0.001
		Steel	65	5.6	0.49	0.22	
		Concrete	32	2.7	0.43	0.19	

Table 2 shows different lifestyle factors associated with the radiation exposure across each age group. Living within 100 meters of a forest was correlated to radiation doses in all age groups (*p*<0.001). However, the participation in an outdoor school sport had a strong correlation with external radiation exposure only for the middle school group (*p*<0.001). In addition, radiation exposure was lower for all three groups when there were other buildings located around the subject’s residence (*p* = 0.001 for the 0-5-year-old group, *p*<0.001 for the elementary and middle school groups).

**Table 2:** Comparison of mean radiation doses across lifestyle factors.

			n	%	Mean (mSv)	SD	*p*-value
**Bedroom floor (n = 4,025)**	0-5-year-olds	1^st^ floor	189	26.7	0.63	0.24	<0.001
		2^nd^ floor	444	62.7	0.68	0.26	
		3^rd^ floor and above	18	2.5	0.46	0.14	
	Elementary school	1^st^ floor	619	26.8	0.67	0.27	<0.001
		2^nd^ floor	1,619	70.0	0.65	0.27	
		3^rd^ floor and above	75	3.2	0.48	0.16	
	Middle school	1^st^ floor	292	27.5	0.64	0.25	0.001
		2^nd^ floor	739	69.7	0.66	0.27	
		3^rd^ floor and above	30	2.8	0.47	0.20	
**Outdoor school sport (n = 3,994)**	0-5-year-olds	Yes	40	6.1	0.65	0.29	0.862
		No	613	93.9	0.66	0.25	
	Elementary school	Yes	490	21.3	0.64	0.30	0.465
		No	1,814	78.7	0.65	0.26	
	Middle school	Yes	507	48.9	0.68	0.27	<0.001
		No	530	51.1	0.62	0.25	
**Do you use wood for the bath or stove? (n = 3,990)**	0-5-year-olds	Yes	74	11.4	0.70	0.37	0.130
		No	574	88.6	0.65	0.24	
	Elementary school	Yes	201	7.7	0.72	0.30	<0.001
		No	2,094	80.7	0.64	0.26	
	Middle school	Yes	105	8.2	0.73	0.28	0.001
		No	942	74.0	0.64	0.26	
**Residence is surrounded by buildings (n = 3,976)**	0-5-year-olds	Yes	401	62.3	0.63	0.24	0.001
		No	243	37.7	0.70	0.28	
	Elementary school	Yes	1,574	60.7	0.62	0.24	<0.001
		No	711	27.4	0.73	0.30	
	Middle school	Yes	693	66.2	0.61	0.24	<0.001
		No	354	33.8	0.72	0.28	
**Forest within 100 meters of residence (n = 3,984)**	0-5-year-olds	Yes	542	84.0	0.68	0.27	<0.001
		No	103	16.0	0.54	0.16	
	Elementary school	Yes	1,871	81.5	0.67	0.28	<0.001
		No	424	18.5	0.55	0.20	
	Middle school	Yes	856	82.0	0.68	0.27	<0.001
		No	188	18.0	0.52	0.19	

The attitude factors in Table 3 were measured by a scale indicative of the guardians’ perceptions towards radiation. In contrast with the 0-5-year-old and elementary group, the middle school group showed strong associations between radiation exposure level and attitude factors. The external radiation dose for the middle school group was statistically significant (*p* = 0.013). Those in the middle school group who owned a radiation measuring device at home showed a lower external radiation dose as compared to those who did not own a measuring device (*p* = 0.004). The middle school participants who indicated that they spent the weekends in low radiation areas (*p* = 0.037) and avoided high radiation places (*p* = 0.029) had a lower mean external radiation dose. Among the middle school participants who indicated they exercised caution towards radiation exposure, the exposure rate decreased as cautiousness increased (b = -0.124, *p* = 0.005). For the middle school group only, the following attitude factors were associated with lower risks of external radiation doses: participation in a study group (b = -0.045, *p*<0.001), owning a radiation measuring device (b = -0.061, *p* = 0.056), level of caution (b = -0.124, *p* = 0.003) and level of concern (b = -0.074, *p* = 0.042) towards radiation.

**Table 3:** Comparison of mean radiation doses across attitude factors.

			n	%	Mean (mSv)	SD	*p*-value
**Did you participate in a study group? (n = 4,002)**	0-5-year-olds	Yes	203	31.2	0.66	0.24	0.711
		No	448	68.8	0.65	0.27	
	Elementary school	Yes	856	37.1	0.64	0.23	0.100
		No	1,454	62.9	0.66	0.29	
	Middle school	Yes	405	38.9	0.62	0.25	0.013
		No	636	61.1	0.66	0.27	
**Do you have a measuring device at home? (n = 4,003)**	0-5-year-olds	Yes	76	10.7	0.66	0.22	0.910
		No	573	80.9	0.66	0.26	
	Elementary school	Yes	300	11.6	0.65	0.33	0.743
		No	2,012	77.5	0.66	0.26	
	Middle school	Yes	133	12.8	0.59	0.23	0.004
		No	909	87.2	0.65	0.26	
**Do you spend the weekend in low radiation areas? (n = 3,996)**	0-5-year-olds	Yes	178	25.1	0.63	0.23	0.097
		No	473	66.8	0.67	0.27	
	Elementary school	Yes	588	22.7	0.64	0.24	0.077
		No	1,717	66.2	0.66	0.28	
	Middle school	Yes	130	12.5	0.60	0.25	0.037
		No	910	87.5	0.65	0.26	
**Do you avoid high radiation areas? (n = 3,990)**	0-5-year-olds	Yes	488	75.2	0.65	0.27	0.788
		No	161	24.8	0.66	0.22	
	Elementary school	Yes	1,717	66.2	0.65	0.27	0.552
		No	584	22.5	0.66	0.26	
	Middle school	Yes	699	67.2	0.63	0.26	0.029
		No	341	32.8	0.67	0.27	
**Level of cautiousness towards radiation (n = 3,999)**	0-5-year-olds	Very much	137	19.4	0.63	0.23	0.016
		A little	439	62.0	0.65	0.26	
		Not so much	66	9.3	0.75	0.28	
		Not at all	8	1.1	0.72	0.30	
	Elementary school	Very much	631	24.3	0.64	0.29	0.148
		A little	1437	55.4	0.66	0.26	
		Not so much	215	8.3	0.65	0.31	
		Not at all	25	1.0	0.76	0.19	
	Middle school	Very much	245	23.5	0.60	0.24	0.005
		A little	658	63.2	0.66	0.26	
		Not so much	120	11.5	0.69	0.31	
		Not at all	18	1.7	0.68	0.26	
**Level of concern towards radiation (n = 3,997)**	0-5-year-olds	Very much	120	16.9	0.67	0.22	0.095
		A little	394	55.6	0.64	0.26	
		Not so much	129	18.2	0.69	0.27	
		Not at all	8	1.1	0.75	0.33	
	Elementary school	Very much	534	20.6	0.65	0.26	0.921
		A little	1355	52.2	0.66	0.29	
		Not so much	382	14.7	0.66	0.24	
		Not at all	35	1.3	0.67	0.22	
	Middle school	Very much	214	20.6	0.63	0.24	0.722
		A little	605	58.2	0.65	0.27	
		Not so much	190	18.3	0.64	0.27	
		Not at all	31	3.0	0.68	0.24	

Table 4 shows the factors associated with external radiation dose stratified by age groups calculated by linear regression. For all age groups, those who had the following characteristics had risks of higher external radiation dose: lived in wooden residences (as compared with steel or concrete residences), had a first floor bedroom (as compared with having a third floor or higher bedroom), had no surrounding buildings around their residence, had a surrounding area that had completed the decontamination process, and had a forest within 100 meters of their residence. Some risk factors were significantly associated with external radiation doses for particular age groups. Among the 0-5-year-old and middle school groups, a high level of cautiousness exercised among the guardians was significantly associated with lower external radiation doses in the children. For the elementary and middle school group, the use of firewood was associated with higher risks of external radiation doses. In addition, middle school students with the following characteristics had lower risks of external radiation doses: participating in a study group, owning a radiation measuring device, spending time in low radiation areas, avoiding high radiation areas, and being concerned about radiation.

**Table 4:** Factors associated with mean radiation doses.

**Factors associated with radiation dose within the 0-5-year-old group**					
**Variables**		**b**	**95% CI**	**S.E.**	***p*-value**
**Type of residence (n = 612)**	Wooden residence (ref)				
	Steel residence	-0.137	(-0.210, -0.057)	0.039	0.001
	Concrete residence	-0.167	(-0.236, -0.085)	0.038	<0.001
**Bedroom floor (n = 615)**	1^st^ Floor (ref)				
	2^nd^ Floor	0.087	(0.004, 0.091)	0.022	0.031
	3^rd^ floor or above	-0.110	(-0.294, -0.049)	0.063	0.006
**Buildings around residences (n = 644)**		-0.066	(-0.107, -0.026)	0.021	0.001
**Forest within 100 m (n = 645)**		0.138	(0.085, 0.191)	0.027	<0.001
**Level of cautiousness (n = 650)**		-0.134	(-0.102, -0.014)	0.022	0.010
**Factors associated with radiation dose within elementary school students**					
**Variables**		**b**	**95% CI**	**S.E.**	***p*-value**
**Type of residence (n = 2,437)**	Wooden residence (ref)				
	Steel residence	-0.119	(-0.175, -0.088)	0.022	<0.001
	Concrete residence	-0.140	(-0.198, -0.112)	0.022	<0.001
**Bedroom floor (n = 2,385)**	1st floor (ref)				
	2nd floor	-0.023	(-0.039, 0.011)	0.013	0.283
	3rd floor or above	-0.124	(-0.254, -0.125)	0.033	<0.001
**Buildings around residences (n = 2,285)**		-0.117	(-0.140, -0.093)	0.000	<0.001
**Forest within 100 m (n = 2,295)**		0.126	(0.098, 0.155)	0.014	<0.001
**Use of firewood (n = 2,295)**		0.073	(0.034, 0.111)	0.020	<0.001
**Factors associated with radiation dose within middle school students**					
**Variables**		**b**	**95% CI**	**S.E.**	***p*-value**
**Type of residence (n = 1,150)**	Wooden residence (ref)				
	Steel residence	-0.151	(-0.240, -0.109)	0.034	<0.001
	Concrete residence	-0.141	(-0.321, -0.136)	0.047	<0.001
**Bedroom floor (n = 1,061)**	1st floor (ref)				
	2^nd^	0.025	(-0.021, 0.049)	0.018	0.431
	3rd floor or above	-0.110	(-0.272, -0.076)	0.050	0.001
**Participation in a school sport (n = 1,037)**		-0.005	(-0.046, 0.036)	0.021	<0.01
**Buildings around residences (n = 1,047)**		-0.104	(-0.137, -0.071)	0.017	<0.001
**Use of firewood (n = 1,047)**		0.090	(0.038, 0.143)	0.027	0.001
**Forest within 100 m (n = 1,044)**		0.154	(0.113, 0.194)	0.021	<0.001
**Study group participation (n = 1,041)**		-0.045	(-0.057, 0.009)	0.017	0.154
**Owning measuring device (n = 1,042)**		-0.061	(-0.097, 0.001)	0.025	0.056
**Spending time in low dose radiation area (n = 1,040)**		-0.018	(-0.066, 0.037)	0.026	0.585
**Avoiding high dose radiation areas (n = 1,040)**		-0.019	(-0.048, 0.027)	0.019	0.583
**Level of cautiousness (n = 1,041)**		-0.124	(-0.084, -0.017)	0.017	0.003
**Level of concernedness (n = 1,040)**		-0.074	(-0.057, -0.001)	0.014	0.042

## Discussion

The present study presents the following key results: external radiation exposure is significantly related to the type of material used for houses, living within 100 meters of a forest, and attitude factors, such as the level of cautiousness towards radiation.

This study found that residences constructed from three different materials (wood, steel, and concrete) had varying levels of external radiation exposure. Wooden residences had higher levels of radiation exposure, as compared to steel and concrete residences [29]. Participants who lived within 100 m of a forest also had higher levels of radiation exposure. In the FDNPP accident, more than 75% of the contaminated area was forested, and the released radioactive elements Cesium-134 and Cesium-137 have half-lives of 2.07 years and 30.1 years, respectively [30, 31]. Further, gamma rays emitted from radioactive materials can travel up to 100 m on the surface of a highly contaminated forested area [32]. Therefore, living in wooden residences and within 100 m of a Fukushima forest can result in higher levels of radiation exposure.

Awareness of radiation was the most common characteristic observed among the attitude factors. Participants who were involved in a radiation study group and owned a radiation measuring device indicated a higher awareness of radiation. For each age group, the mean radiation dose tended to decrease as awareness increased. Similarly, the lack of awareness and concern regarding radiation could have been associated with increased exposure to external radiation. For example, after the Chernobyl accident, some residents returned to their highly contaminated homes because they did not have sufficient knowledge on the risks of radiation [33]. Therefore, increasing awareness of radiation can potentially reduce adverse effects of radiation exposure. Increasing awareness and knowledge of radiation could also prevent the development of long-term psychological effects among affected individuals. With the high prevalence of chronic mental illnesses from nuclear disasters, educating students about nuclear radiation would be an effective method of reducing these risks.

The present study also found that radiation exposure doses were related to participation in outdoor activities. Children in the middle school group who participated in outdoor school sports had higher levels of external radiation exposure, which could be attributed to differences in indoor and outdoor radiation concentrations. In Japan, schools are typically built from concrete, creating a stronger shield from radiation. Thus, when in school, students are less likely to be exposed to radiation. However, participating in outdoor activities can increase the students’ radiation exposure. In Fukushima, some schools restrict the amount of time children spend outdoors to less than 3 hours a day [34]. This finding is supported by existing literature on the association between outdoor activity and external radiation exposure. Yet, limiting the amount of outdoor activities can adversely affect student health, potentially increasing the risk of obesity, diabetes, and other metabolic health issues [35]. This is the first study to collectively explore the lifestyle factors associated with external radiation exposure among a large number of children living in Nihonmatsu City of the Fukushima Prefecture. Thus, with little previous knowledge and awareness of the health effects of radiation on the residents’ lives, this study helps elucidate the different factors associated with individual external radiation exposure.

The present study had several limitations. First, the results of this study may not be applicable to other municipalities. The association between external radiation dose and lifestyle factors may differ between geographical regions. Second, the study was limited by the investigated age groups. By focusing only on the guardians’ attitudes, there is an underrepresentation of the students’ perspectives toward radiation. Third, participants voluntarily answered the questionnaires and wore the badges. Therefore, participants may have been more cautious in their answers and actions than those who did not participate in the study. Fourth, the self-reported questionnaires may have caused some unintentional bias, leading to the possible overestimation or underestimation of the results and causing some participants to answer positivity because of the city’s involvement. Finally, there may be measurement errors as a result of the radiation badges. The analysis used adjusted radiation doses, where the natural environmental background dose was subtracted, but this method assumes a linear decay. The radiation badges provide limited information concerning the location of exposure. Therefore, the amount of exposure from each location, as well as how much radiation is shielded from each location or residence, is uncertain.

## Conclusion

This study stresses the importance of radiation protection to prevent adverse health effects in the future. Lifestyle factors, such as living in wooden residences and living within 100 m of a forest, are associated with higher external radiation exposure. Conversely, attitude factors towards radiation were correlated with lower radiation exposure. These results indicate a need for improved and continuous radiation awareness and education. This study is one of the first studies to shed light on the importance of guardian attitudes in the radiation exposure rate of children. In addition, this study provides valuable insight into the importance of attitude towards and awareness of radiation. Therefore, in the event of future radiation disasters, attitudes towards and awareness of radiation should be considered in the reduction of exposure risk and implementation of radiation protection measures.
